# Hemoglobin and B-type natriuretic peptide preoperative values but not inflammatory markers, are associated with postoperative morbidity in cardiac surgery: a prospective cohort analytic study

**DOI:** 10.1186/1749-8090-8-170

**Published:** 2013-07-05

**Authors:** Edgar Hernández-Leiva, Rodolfo Dennis, Daniel Isaza, Juan Pablo Umaña

**Affiliations:** 1 Department of Cardiology, Section of Cardiovascular Critical Care, Instituto de Cardiología-Fundación Cardioinfantil. Universidad del Rosario, Bogotá, Colombia; 2 Department of Internal Medicine, Instituto de Cardiología-Fundación Cardioinfantil, Bogotá, Colombia; 3 Department of Cardiology, Section of Coronay Care, Instituto de Cardiología-Fundación Cardioinfantil, Bogotá, Colombia; 4 Department of Cardiovascular Surgery, Instituto de Cardiología-Fundación Cardioinfantil, Bogotá, Colombia

**Keywords:** Cardiac surgery, Hemoglobin, Inotropic agents, Natriuretic peptides, Postoperative care

## Abstract

**Introduction:**

Risk stratification in cardiac surgery significantly impacts outcome. This study seeks to define whether there is an independent association between the preoperative serum level of hemoglobin (Hb), leukocyte count (LEUCO), high sensitivity C-reactive protein (hsCRP), or B-type natriuretic peptide (BNP) and postoperative morbidity and mortality in cardiac surgery.

**Methods:**

Prospective, analytic cohort study, with 554 adult patients undergoing cardiac surgery in a tertiary cardiovascular hospital and followed up for 12 months. The cohort was distributed according to preoperative values of Hb, LEUCO, hsCRP, and BNP in independent quintiles for each of these variables.

**Results:**

After adjustment for all covariates, a significant association was found between elevated preoperative BNP and the occurrence of low postoperative cardiac output (OR 3.46, 95% CI 1.53–7.80, p = 0.003) or postoperative atrial fibrillation (OR 3.8, 95% CI 1.45–10.38). For the combined outcome (death/acute coronary syndrome/rehospitalization within 12 months), we observed an OR of 1.93 (95% CI 1.00–3.74). An interaction was found between BNP level and the presence or absence of diabetes mellitus. The OR for non-diabetics was 1.26 (95% CI 0.61–2.60) and for diabetics was 18.82 (95% CI 16.2–20.5). Preoperative Hb was also significantly and independently associated with the occurrence of postoperative low cardiac output (OR 0.33, 95% CI 0.13–0.81, p = 0.016). Both Hb and BNP were significantly associated with the lengths of intensive care unit and hospital stays and the number of transfused red blood cells (p < 0.002). Inflammatory markers, although associated with adverse outcomes, lost statistical significance when adjusted for covariates.

**Conclusions:**

High preoperative BNP or low Hb shows an association of independent risk with postoperative outcomes, and their measurement could help to stratify surgical risk. The ability to predict the onset of atrial fibrillation or postoperative low cardiac output has important clinical implications. Our results open the possibility of designing studies that incorporate BNP measurement as a routine part of preoperative evaluation, and this strategy could improve upon the standard evaluation in terms of reducing adverse postoperative events.

## Background

Cardiac surgery is associated with significant risk of morbidity and mortality. In-hospital mortality of patients undergoing myocardial revascularization surgery is approximately 2.5%, and major morbidity events are reported in 22% of cases [1]. Risk stratification plays an important role in this type of surgery, and various risk models are used in clinical practice. Currently, two of the most used models are the EuroSCORE (The European System for cardiac operative Risk Evaluation) and STS (The Society of Thoracic Surgeons National Cardiac Database). These models estimate postoperative mortality considering patient and procedural factors; however, their application has some complexity, and the information needed to calculate the scores is not always available. EuroSCORE, especially, tends to overestimate mortality [2-4]. Moreover, although several risk factors are associated with poor prognosis after surgery, including advanced age, preoperative functional capacity, renal failure, diabetes mellitus, emergency surgery, and decreased ventricular function, these well-defined factors for increased risk are not modifiable; consequently, there is little that can be done to control them [5-7].

The identification of laboratory measurements or preoperative serum markers that are independently associated with postoperative adverse outcomes could be the initial step in the eventual inclusion of these variables in risk prediction models. Among these biomarkers, a slightly lower-than-normal hemoglobin (Hb) level has been identified as an independent predictor of poor functional capacity and mortality in patients with congestive heart failure [8]. Similarly, in coronary patients undergoing percutaneous coronary intervention or cardiac surgery, such biomarkers have been associated with a poor prognosis [9,10]. Because low Hb is a potentially modifiable factor, its identification in patients with coronary or valvular heart disease, or who are to undergo elective surgery, would eventually help to design therapeutic regimens designed to correct the Hb level and then assess the impact of this treatment on postoperative outcomes.

Furthermore, several inflammatory markers, including high-sensitivity C-reactive protein (hsCRP) and leukocyte count (LEUCO), have been suggested as independent predictors of future cardiovascular risk in non-surgical patients [11,12]. Despite the important role of inflammation in the pathophysiology of cardiovascular disease, few studies with an adequate number of patients have evaluated the relationships of preoperative hsCRP and LEUCO values with postoperative outcomes.

B-type natriuretic peptide (BNP) is an important prognostic indicator in both heart failure and acute myocardial infarction, where elevated levels identify patients at high risk for progressive ventricular dilatation, heart failure, or death. [13-15]. BNP is released in response to ventricular overload and in myocardial ischemia as a result of increased wall stress [16]. The ability of BNP to integrate the effects of various manifestations of myocardial dysfunction makes it useful as an indicator of cardiovascular prognosis in many contexts. Perioperative BNP determination could be useful in predicting some adverse outcomes after cardiac surgery [17,18]. These findings are generally found in small retrospective series and require confirmation in prospective cohorts and larger studies.

This study attempts to determine if there is an independent association between the preoperative serum level of Hb, LEUCO, hsCRP, or BNP and early and late postoperative morbidity. A valuable contribution to the preoperative evaluation could be gained by defining a quantitative and cost-effective serum marker that shows a demonstrated a significant independent association with variables such as mortality and postoperative low cardiac output (PLCO). Moreover, surgical mortality is often analyzed as in-hospital mortality or at 30 days, which reflects only part of the overall outcome after cardiac surgery. Patients with complicated stays in the intensive care unit (ICU) may persist with limited functional capacity, readmissions, and shorter survival time after being discharged, so an assessment of longer-term morbidity and mortality could provide a more reliable analysis of the benefit of cardiac surgery to patients.

## Methods

The study included all adult patients who underwent elective cardiac surgery in a tertiary cardiovascular hospital during a period of 18 months. Subjects who had emergency surgery, maze surgery, atrial septal closure, chronic obstructive pulmonary disease, any clinically important bleeding in the last 6 months, recent blood transfusion (less than 2 weeks), or renal failure were excluded. Also excluded were patients with LEUCO < 2500 mm^3^ or > 30,000 mm^3^ or who had active infectious, inflammatory, or neoplastic diseases. The reasons for excluding these subgroups are summarized as follows: Emergency surgery is always a high-risk procedure and would introduce difficult-to-control variables into the study. In maze surgery, there are frequent postoperative rhythm disorders, and hospital stays are usually longer; thus, many of these outcome variables would be altered for reasons intrinsic to the surgical procedure. In atrial septal closures, the possibility of complications is very low, and this subgroup of patients would not provide information to the study; pulmonary disease and chronic kidney disease alter hemoglobin, and both can raise BNP, which are the exposure variables being evaluated. Similarly, patients with diseases that could alter inflammatory markers were excluded.

### Study design and variables

The cohort was distributed according to Hb, LEUCO, hsCRP, and BNP in separate quintiles for each variable. All patients were followed up for 12 months from the date of surgery. Pre-specified definitions were used to assign background or outcomes.

The primary objective was to determine whether there is an association between the preoperative value of any of these biomarkers and morbidity and mortality in the first 12 months postoperatively. The most important event for the study (primary outcome) was a combination of death/myocardial infarction/cumulative rehospitalization at 12 months postoperatively. Taking anesthetic induction as the starting time, all clinically relevant outcomes were recorded: postoperative bleeding requiring reoperation, death from any cause during hospitalization, prolongation of stay in the ICU or the hospital, number of red blood cells transfused postoperatively, lowest postoperative Hb level, surgical wound infection, PLCO, acute renal failure, cerebrovascular events, atrial fibrillation (AF), ventricular tachycardia or ventricular fibrillation, perioperative myocardial infarction, and use of inotropes (over 24 hours) postoperatively. All events that took place from the time of hospital discharge until the last day of the 12-month follow-up (death from any cause, rehospitalization, acute coronary syndrome (ACS), cerebrovascular events, or further revascularization procedures or valve repair) were also recorded. This protocol of research was presented to the ETHICS COMMITTEE OF CLINICAL INVESTIGATION, Instituto de Cardiología-Fundación Cardioinfantil and approved for its execution.

### Statistical methods

Because the defined primary endpoint was the estimation of the significance and magnitude of the association between each of the exposure variables and the outcome variable, we decided that these significance values could be ascertained by multiple regression or logistic analysis more than by analysis of survival or time to event.

Both the differences in basic clinical characteristics between quintiles and the unadjusted relationship between each sequence of preoperative quintiles and the specified primary and secondary outcomes were assessed using the trends test developed by Cuzick. Potential preoperative confounders were included as covariates to be analyzed using regression models of association. The association between the primary dependent variable and the independent variables was assessed, adjusting for covariates by logistic regression analysis. Subsequently, the association between each of the secondary dependent variables and independent variables was assessed, adjusting for covariates.

It was modeled by means of logistic regression of association (evaluating interaction) for dichotomous outcomes. In the multivariate model all the covariables were included together with the exposure variables in accordance with the concept proposed by Kleinbaum [19] of which the best model (if not the most parsimonious) is the one that controls for all the confusion variables. OR and confidence intervals of 95 % were calculated for each of the outcomes.

Because the sample was classified into quintiles, reported ORs were for the expected increase in risk between the two extreme quintiles (e.g., lowest BNP vs. highest BNP), taking as comparator the first quintile of BNP (containing the lowest BNP values) or the fifth quintile of Hb (containing the highest Hb values); the second, third, and fourth quintiles were used in the description of the raw data and the association of each independent variable to each of the outcomes, but not in the regression analysis. The variables were defined as follows:

•Independent variables: Hb, LEUCO, hsCRP, and BNP.

•Primary dependent variable: Death/MI/rehospitalization

•Secondary dependent variables: All outcomes described.

•Covariates: Age, left ventricular ejection fraction, gender, diabetes mellitus, obesity, ACS in the last month, previous cardiac surgery, type of surgery (1 = myocardial revascularization, 2 = valve replacement, 3 = myocardial infarction revascularization + valve replacement with or without an associated procedure), and use or non-use of cardiopulmonary bypass.

A separate model excluding 16 subjects with baseline (preoperative) Hb > 17.5 g/dL was prepared to evaluate the hypothesis that the risk associated with Hb had a "U" morphology in which risk also increased at high Hb values.

For all tests, p values < 0.05 were considered significant. All analyses were performed in Stata 8.0

## Results

### Baseline characteristics of the cohort

**Figure 1:**
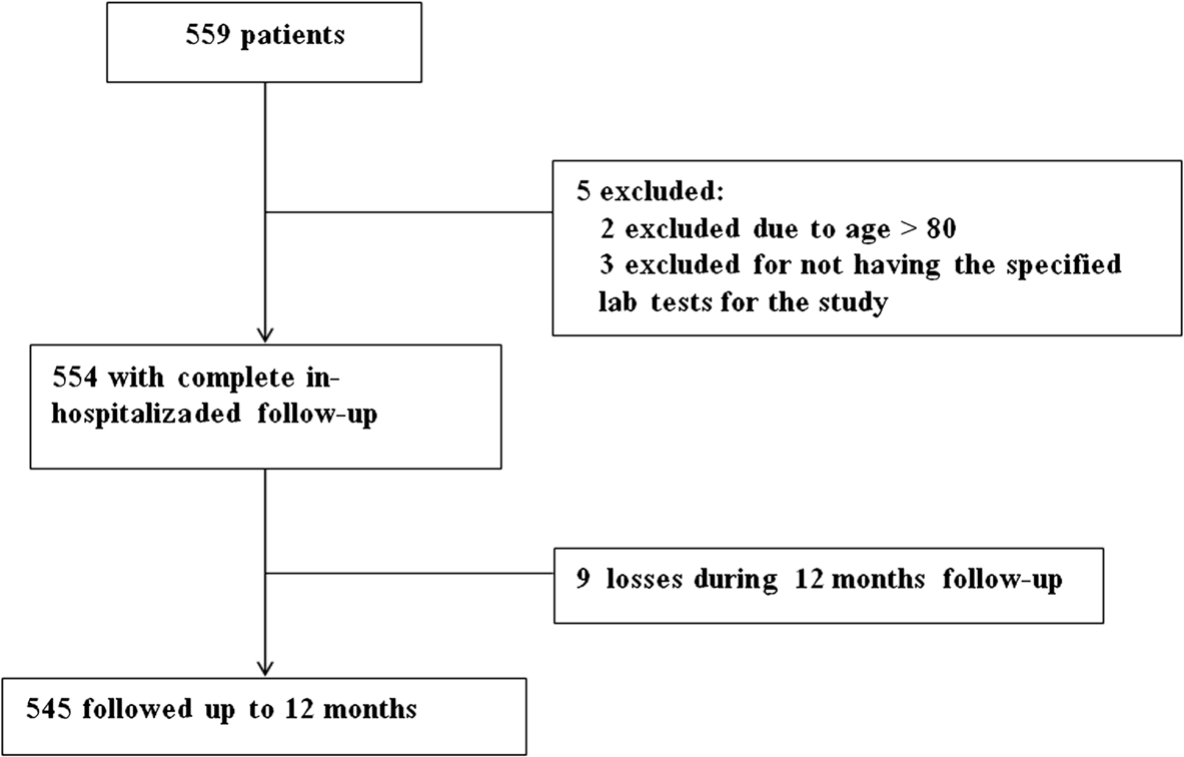
Flowchart.

A total of 559 patients were included, of whom 545 (97%) had a complete follow-up of 12 months. See Figure 1*.* The baseline characteristics of the patients are shown in Table 1.

**Table 1:** Baseline characteristics of the cohort (n = 554)

Age: mean ± SD	60 ±11
Age ≥ 65: n (%)	209 (37)
Males: n (%)	409 (73.8 )
Residents in a city with an altitude of 2000 - 3000 mts: n (%)	455 (82.1)
Ejection Fraction: mean % ± SD	52.1 ± 11
Diabetes Mellitus: n (%)	111 (20)
Acute Coronary Syndrome ≤ 30 days: n (%)	189 (34.1)
Obesity: n (%)	99 (17.8)
Previous Cardiac Surgery: n (%)	36 (6.5 )
Proportion of Patients undergoing CABG: n (%)	387 (69.86)
Surgery without extracorporeal circulation: n (%)	126 (22.7)

Tables 2, 3, 4 and 5 summarize the characteristics of patients according to the values (distributed in quintiles) of Hb, LEUCO, hsCRP, and BNP. Preoperative Hb was lower in women (p < 0.001) and decreased with age (p < 0.001). Patients who were residents of cities located at an altitude of 2000-3000 m had higher Hb (p = 0.01), and those with a history of ACS in the last month had lower Hb (p = 0.01).

**Table 2:** Baseline characteristics according to preoperative hemoglobin value

**Hemoglobin (g/dL)**	**Quintile 1 (8.8–13.2) (n = 114)**	**Quintile 2 (13.3-14.2) (n =113)**	**Quintile 3 (14.3-15.1) (n =118)**	**Quintile 4 (15.2 – 16) (n = 108)**	**Quintile 5 (16.1-20.2) (n = 101)**	**Trend test**
Mean age (SD)	62.3 (11.9)	61.6 (10.9)	59.9 (11.2)	58.7 (9.3)	57.3 (11.9)	P = 0.001
Males (%)	37.7	63.7	78.8	95.3	97.0	P = 0.001
Residents in a city with an altitude of 2000 to 3000 m (%)	72.8	84.0	83.9	82.4	88.1	P = 0.01
Mean ejection fraction (%)	50.7	53.3	52.4	52.6	51.3	P = 0.89
Diabetes mellitus (%)	25.4	17.7	18.6	17.6	20.7	P = 0.4
Acute coronary syndrome ≤ 30 days (%)	44.7	34.5	32.2	30.5	27.7	P = 0.01
Obesity (%)	14.9	17.7	16.1	20.3	20.8	P = 0.22
Previous cardiac surgery (%)	6.1	4.4	10.1	3.7	7.9	P = 0.71
Proportion of patients undergoing CABG (%)	68.4	67.2	71.2	72.2	70.3	P = 0.09

**Table 3:** Baseline characteristics according to preoperative B-type natriuretic peptide

**B-type natriuretic peptide**	**Quintile 1 (0 - 25.2) (n = 111)**	**Quintile 2 (25.3 – 53.6 ) (n =111)**	**Quintile 3 (53.7 – 108.3) (n =111)**	**Quintile 4 (109.2 – 240.4) (n = 111)**	**Quintile 5 (258 - 4000) (n = 110)**	**Trend test**
Mean age (SD)	59.0 (9.7)	57.5 (11.4)	60.8 (12.1)	61.2 (11.3)	61.5 (11.0)	P=0.001
Males (%)	78.3	76.5	71.1	68.4	74.5	P = 0.23
Residents in a city with an altitude of 2000- 3000 m (%)	85.6	81.0	80.1	81.0	82.7	P = 0.64
Mean ejection fraction (%)	54.0 (9.2)	53.8 (11.3)	54.2 (11.1)	51.2 (11.0)	45.1 (12.7)	P = 0.001
Diabetes mellitus (%)	23.4	18.9	15.3	17.1	25.4	P = 0.86
Acute coronary syndrome ≤ 30 days (%)	14.4	35.1	31.5	42.3	47.2	P = 0.001
Obesity (%)	25.2	16.2	19.8	17.1	10.9	P = 0.02
Previous cardiac surgery (%)	5.4	6.3	8.1	5.4	7.2	P = 0.7
Proportion of patients undergoing CABG (%)	72.9	79.2	68.4	71.1	57.2	P = 0.001

**Table 4:** Baseline characteristics according to preoperative high-sensivity c-reactive protein

**Hs C-reactive protein**	**Quintile 1 (0.02 - 0.19) (n = 114)**	**Quintile 2 (0.2 – 0.42 ) (n =111)**	**Quintile 3 (0.43 – 0.78) (n =108)**	**Quintile 4 ( 0.79 – 2.14) (n = 111)**	**Quintile 5 (2.15 - 16) (n = 110)**	**Trend test**
Mean age (SD)	58.7 (11.3)	57.9 (12.5)	60.1 (10.9)	61.4 (9.7)	61.9 (10.8)	P = 0.01
Males (%)	74.5	71.1	68.5	78.3	76.3	P = 0.42
Residents in a city with an altitude of 2000- 3000 m (%)	75.4	79.2	87.9	81.9	86.3	P = 0.03
Mean ejection fraction (%)	51.8 (12.3)	54.9 (11.1)	52.3 (11.9)	50.7 (11.5)	50.7 (11.5)	P = 0.06
Diabetes mellitus (%)	19.3	22.5	20.3	19.8	18.1	P = 0.69
Acute coronary syndrome ≤ 30 days (%)	10.5	18.9	30.5	51.3	60.0	P < 0.001
Obesity (%)	11.4	23.4	22.2	19.8	12.7	P = 0.96
Previous cardiac surgery (%)	7.0	9.9	6.4	6.3	2.7	P = 0.10
Proportion of patients undergoing CABG (%)	59.6	62.1	72.2	77.4	78.1	P < 0.001

**Table 5:** Baseline characteristics according to preoperative leukocyte count

**Leukocyte couNT**	**Quintile 1 (3100 – 5600) (n = 111)**	**Quintile 2 (5620 – 6500) (n =114)**	**Quintile 3 (6510 – 7320) (n =108)**	**Quintile 4 (7370 – 8580) (n = 111)**	**Quintile 5 (8590 – 16200) (n = 110)**	**Trend test**
Mean age (SD)	61.7 (11.2)	60.6 (11.5)	59.5 (11.4)	59.8 (11.0)	58.4 (10.7)	P = 0.01
Males (%)	67.5	71.9	79.6	77.4	72.7	P = 0.23
Residents in a city with an altitude of 2000- 3000 m (%)	90	85.9	85.2	74.7	74.5	P < 0.001
Mean ejection fraction (%)	53.3 (10.3)	52.9 (11.8)	52.4 (11.4)	50.2 (12.6)	51.6 (12.1)	P = 0.2
Diabetes mellitus (%)	19.8	15.8	17.8	18.9	28.2	P = 0.10
Acute coronary syndrome ≤ 30 days (%)	42.3	26.3	32.4	34.2	35.4	P = 0.69
Obesity (%)	15.3	14.0	23.1	18.9	18.1	P = 0.35
Previous cardiac surgery (%)	5.4	7.0	7.4	5.4	7.2	P = 0.78
Proportion of patients undergoing CABG (%)	69.3	62.2	67.6	72.9	77.2	P = 0.07

Serum hsCRP increased significantly with age (p = 0.01), the altitude of residence (p = 0.03), history of ACS (p = 0.001), and the diagnosis of coronary artery disease (p = 0.001).

Serum BNP increased significantly with age (p = 0.001), the presence of coronary artery disease (p = 0.001), and a history of recent ACS (p = 0.001). Serum BNP decreased as ejection fraction increased (p = 0.001) and in obese patients (p = 0.001). LEUCO decreased with age (p = 0.01) and in subjects residing at an altitude of 2000-3000 m (p = 0.001).

### Outcomes

**Figure 2:**
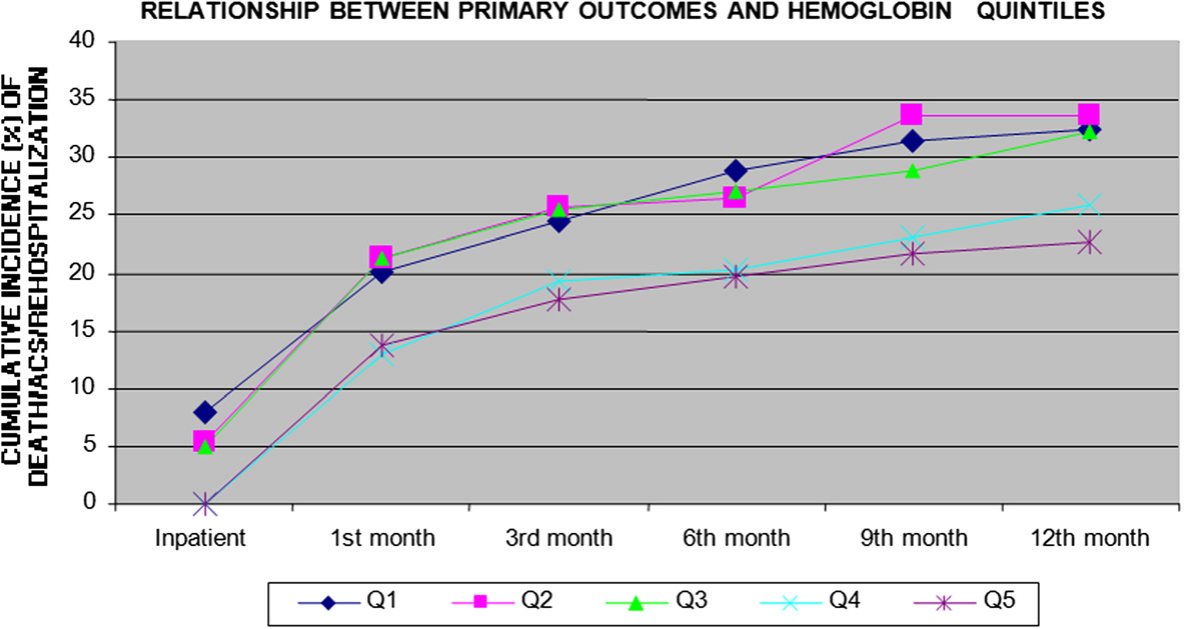
Cumulative incidence of the combined outcome (death/ACS/rehospitalization) for hemoglobin quintiles through 12 months.

**Figure 3:**
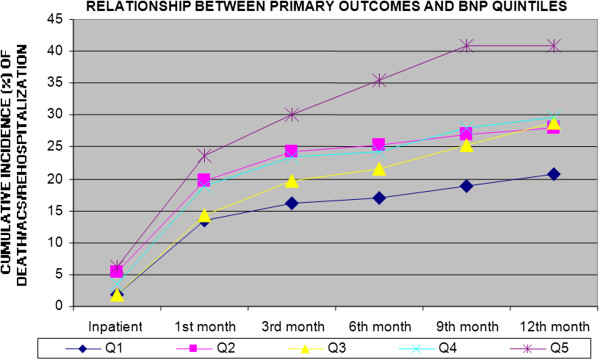
Cumulative incidence of the combined outcome (death/ACS/rehospitalization) for B-Type Natriuretic Peptide (BNP) quintiles through 12 months.

**Figure 4:**
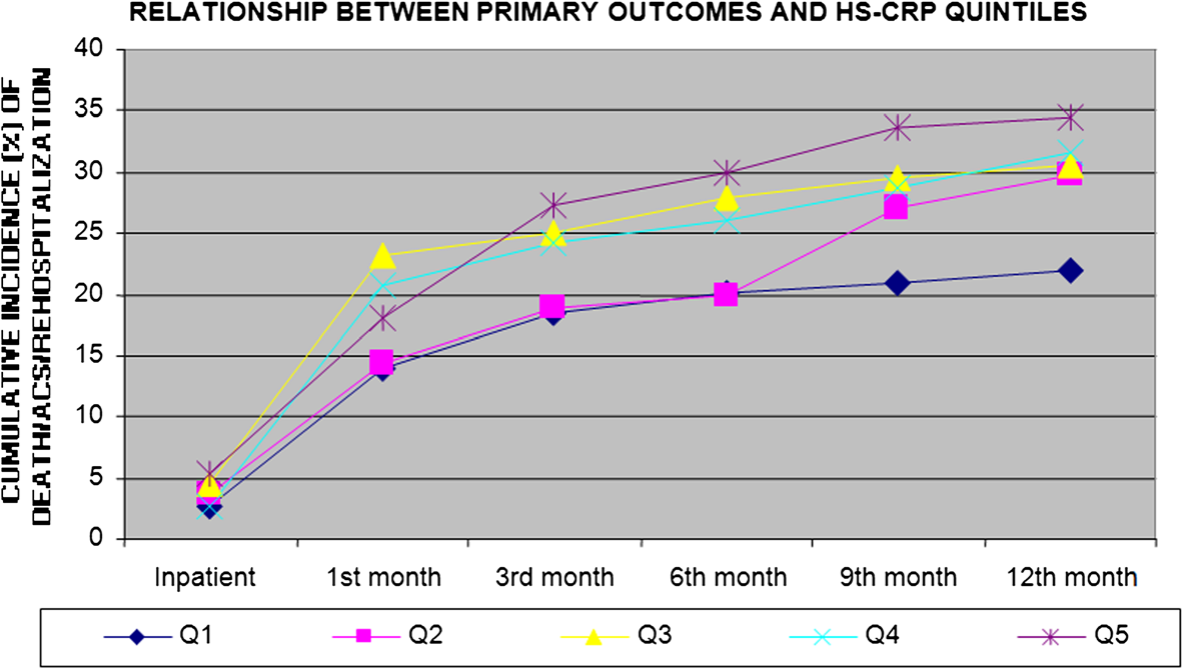
Cumulative incidence of the combined outcome (death/ACS/ rehospitalization) for hs C-Reactive Protein (hs-CRP) quintiles through 12 months.

**Figure 5:**
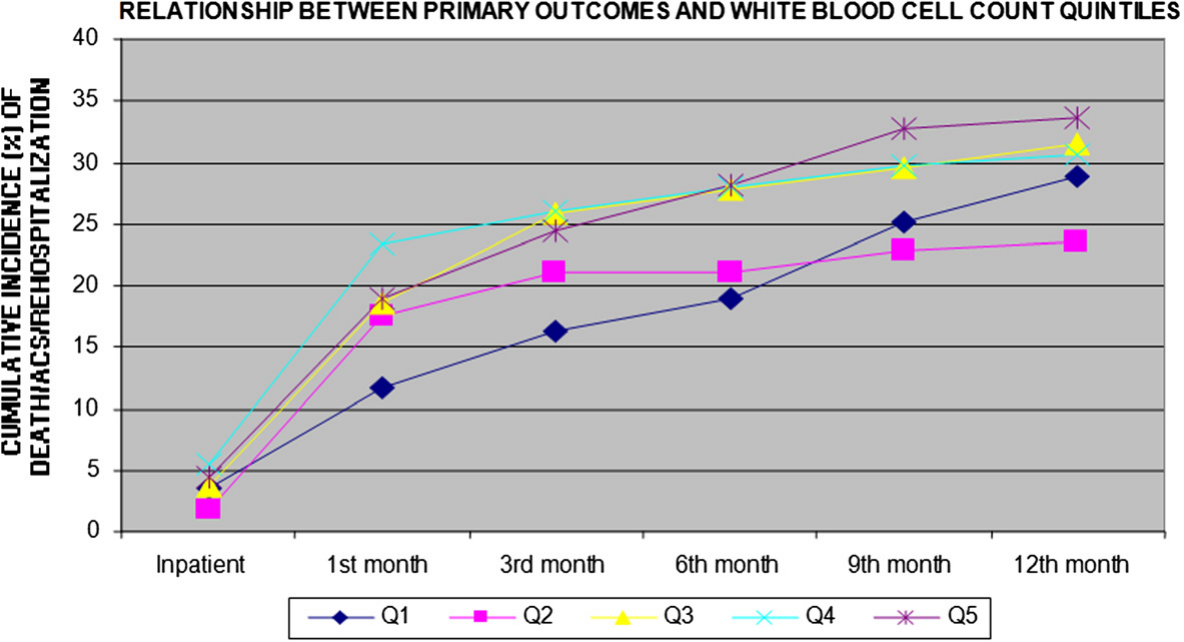
Cumulative incidence of the combined outcome (death/ACS/rehospitalization) for white blood cell count quintiles through 12 months.

Figures 2, 3, 4 and 5 show the incidence of the outcome death/ACS/ rehospitalization from the inpatient period and throughout the follow-up to 12 months, for each of the variables Hb, LEUCO, hsCRP, and BNP. We observed that the increases in BNP and hsCRP were associated with adverse outcomes. For BNP, the cumulative incidence of the composite outcome at 12 months was 20.7% in the lowest quintile, 27.9% in the second quintile, 28.8% in the third quintile, 29.7% in the fourth quintile, and 40.9% in the highest quintile (p = 0.002). The corresponding values for CRP were 21.9%, 29.7%, 30.5%, 31.5%, and 34.5% (p = 0.047). No association with adverse outcomes was observed for the other two variables: Hb: 32.4%, 32.6%, 32.2%, 25.9%, and 22.7% (p = 0.055); LEUCO: 28.8%, 23.6%, 31.4%, 30.6%, and 33.6% (p = 0.22).

In-hospital mortality was 2.7% (15 subjects) (Table 6). The crude death rate was higher in patients with higher values of BNP and hsCRP, but these associations in the unadjusted data were not evaluated statistically because of the low incidence of in-hospital mortality.

**Table 6:** Inhospital outcomes (n = 554)

Overall mortality. n(%)	15 (2.7)
Surgical reintervention. n(%)	39 (7)
Postoperative low cardiac output (PLCO). n(%)	109 (19.6)
Postoperative acute renal failure. n(%)	8 (1.4)
Stroke. n(%)	13 (2.3)
Perioperatory Acute Myocardial Infarction. n(%)	9 (1.6)
Postoperative Atrial Fibrillation. n(%)	62 (11.2)
Postoperative Ventricular Tachycardia. n(%)	16 (2.9)
Intensive Care Unit (ICU) stay (days). Median (range)	1 (1-34)
Hospital stay (days). Median (range)	5 (1-42)
Units of transfused red blood cells (RBC) (intra + post-operative). mean (SD)	1.5 (SD: 2.6)
Lowest postoperative hemoglobin value. mean (SD)	10.4 (SD: 1.4)

A total of 109 patients (19.6%) had a PLCO (Table 6). BNP showed a direct linear relationship with the occurrence of PLCO: 9.9% of subjects in the first quintile compared with 11.7% in the second quintile, 15.3% in the third quintile, 17.1% in the fourth quintile, and 44.5% in the fifth quintile (p = 0.0001).

The incidence of postoperative AF was 11.2% (62 patients). A linear association between preoperative BNP concentration and the occurrence of this complication was found: 6.3% of AF in the bottom BNP quintile vs. 20.9% in the highest quintile (p = 0.01).

The type of surgery was strongly associated with all early outcomes; the mortality rate for group 1 (myocardial revascularization) was 1.5%, 1.2% for group 2 (valve), and 17.2% for group 3 (combined procedures) (p = 0.0001).

Analysis of the unadjusted data showed that hsCRP increased significantly with age and with a history of a recent ACS. When assessed by type of surgery, mean hsCRP was higher in patients undergoing coronary artery bypass: 1.63 mg/L in isolated coronary revascularization, 2.29 mg/L in coronary artery bypass combined with valve surgery, and 0.75 mg/L in valve surgery (p = 0.02). As shown in Figure 4, when evaluating raw data, hsCRP level exhibited a linear relationship with the primary outcome.

The total number of transfused red blood cells (RBC) (intraoperative + postoperative) was 1.5 units on average (SD: 2.6), and this value showed an inverse linear association with preoperative Hb. The number of transfused RBC units followed a linear trend with Hb quintiles: 3.1 (SD: 3.5) for the lowest quintile, 1.7 (SD: 2.8) for the second quintile, 1.4 (SD: 2.1) for the third quintile, 0.7 (SD: 1.3) for the fourth quintile, and 0.6 (SD: 1.6) for the highest quintile (p = 0.0001).

The median ICU stay was 1 day (range: 1-34 days). The duration of hospitalization for qualifying surgery had a median of 5 days (range: 1-42 days). (Table 6)**.** When comparing each of these times between the first and fifth quintiles, a statistically significant difference (p = 0.00001) was found, and also for the comparisons between extreme Hb quintiles and the length of ICU stay (p = 0.002) and length of hospital stay (p = 0.0088). There was no association between these dependent variables and inflammatory markers, hsCRP (p = 0.46), or LEUCO (p = 0.98)

Nonparametric tests showed an unadjusted significant association between high BNP (fifth quintile) and total RBC units transfused in the perioperative period (p = 0.0001), and between low hemoglobin (first quintile) and total RBC units transfused perioperatively (p = 0.00001). There was no association between red blood cells units and LEUCO (p = 0.29) or hsCRP (p = 0.63).

Follow-up through 12 months was achieved in 98.4% of the patients. During this time, 164 primary endpoint events occurred in 107 subjects (cumulative death/ACS/reshopitalization) Cumulative overall mortality at 12 months was 4.15%. The raw data showed a direct linear relationship between each of these factors and the outcome: p = 0.001 for Hb, p = 0.01 for BNP, p = 0.03 for WBC, and p = 0.09 for hsCRP. There were 143 rehospitalization events (25.81%). A significant, direct linear relationship was found between this outcome and BNP level (p = 0.01).

### Multivariate analysis

After adjustment for all covariates described, the ORs for all primary and leading secondary outcomes are shown in Tables 7, 8, 9, 10 and 11. The highest quintile (or lowest in the case of Hb) was used as a comparator for all regression analyses. The reported ORs refer to comparisons with the first quintile (or fifth in the case of Hb). The specific values of the quintiles for each variable are described in Tables 2, 3, 4 and 5.

**Table 7:** Multivariate hemoglobin analysis

**Outcomes**	**Adjusted OR**	**95% CI**
Primary (death/ACS/rehospitalization through 12 months)	0.88	0.43 – 1.78
Atrial fibrillation	0.75	0.28 – 2.01
Perioperatory stroke	0.45	0.038 – 5.29
Reintervention required	0.86	0.28 – 2.62
Low cardiac output syndrome	0.33	0.13 – 0.81
Ventricular tachycardia	0.10	0.01 – 1.10

**Table 8:** Multivariate leukocyte count analysis

**Outcomes**	**Adjusted OR**	**95% IC**
Primary (death/ACS/rehospitalization through 12 months)	1.41	0.78 – 2.55
Atrial fibrillation	1.19	0.50 – 2.81
Perioperatory stroke	1.19	0.15 – 9.04
Reintervention required	0.81	0.25 – 2.58
Low cardiac output syndrome	0.73	0.35 – 1.51
Ventricular tachycardia	0.80	0.22 – 2.89

**Table 9:** Multivariate high-sensivity CRP analysis

**Outcomes**	**Ajusted OR**	**95% IC**
Primary (death/ACS/rehospitalization through 12 months)	1.53	0.80 – 2.91
Atrial fibrillation	0.76	0.31 – 1.89
Perioperatory stroke	0.26	0.033 – 2.09
Reintervention required	0.72	0.20 – 2.57
Low cardiac output syndrome	1.55	0.73 – 3.30
Ventricular tachycardia	2.26	0.15 – 33.28
Primary (death/ACS/rehospitalization through 12 months)	1.23	0.24 – 6.17

**Table 10:** Type-b natriuretic peptide interaction with diabetes mellitus (multivariate analysis)

**Outcomes**	**Adjusted OR DM (-)**	**95% CI**	**Adjusted OR DM (+)**	**95% CI**
Primary (death/ACS/rehospitalization through 12 months)	1.26	0.61–2.61	18.8	16.2 – 20.5
Rehospitalization through 12 months	1.21	0.57–2.56	18.7	16.5 – 20.8

**Table 11:** Type-b natriuretic peptide (multivariate analysis)

**Outcomes**	**OR adjusted**	**95% IC**
Atrial fibrillation	3.80	1.45 – 10.38
Perioperatory cerebrovascular event	1.96	0.18 – 21.40
Need for reintervention	0.88	0.31 – 2.49
Low cardiac output syndrome	3.46	1.53 – 7.80
Ventricular tachycardia	1.74	0.34 – 8.82

### Hemoglobin

The OR for the composite primary outcome at 12 months was 0.88 p = 0.72 (95% CI 0.43-1.78). The adjusted OR for in-hospital mortality was 0.9 (95% CI 0.24–3.30) and for 12-month mortality was 0.20 (95% CI 0.02–1.89). Preoperative Hb level was significantly and independently associated with the occurrence of PLCO (OR 0.33, 95% CI 0.13–0.81, p = 0.016).

The occurrence of postoperative ventricular tachycardia was highly related to in-hospital mortality; mortality in the group that suffered this condition was 18.7% vs. 2.2% in the group without this arrhythmia (p = 0.0001). Preoperative Hb showed a marginal association with the emergence of VT, with an estimated OR of 0.10 (95% CI 0.01–1.10). Other outcomes were rare and showed no statistically significant association with mortality.

Separate analysis of patients with Hb > 17.5 g/dL found no evidence of increased risk in this subgroup of patients.

### Leukocyte count

There was no significant independent association between LEUCO and any of the variables studied. The adjusted OR for the primary outcome was 1.41 (95% CI 0.78–2.55), for in-hospital mortality 3.19 (95% CI 0.47–21.5), and mortality at 12 months 3.73 (95% CI 0.88–15.85). For cumulative rehospitalization at 12 months, the OR was 1.18 (95% CI 0.64–2.18). The association between LEUCO and cumulative ACS at 12 months had an OR of 1.62 (95% CI 0.56–4.72).

### High-sensitivity CRP

hsCRP had no significant independent association with any of the variables studied. The OR for the composite primary outcome at 12 months was 1.53 (95% CI 0.80–2.91). The adjusted OR for in-hospital mortality was 2.1 (95% CI 0.20 – 21.96) and for 12-month mortality was 3.28 (95% CI 0.34–31.7). For cumulative rehospitalization at 12 months, the OR was 1.43 (95% CI 0.73–2.76). For cumulative ACS at 12 months, the OR was 2.33 (95% CI 0.66–8.28). hsCRP also had no independent significant association with other outcome variables, such as PLCO, AF, cerebrovascular events, ventricular tachycardia, and acute renal failure.

### B-type natriuretic peptide

The OR for the association between BNP and the composite primary outcome at 12 months was 1.93 (95% CI 1.00–3.74). However, a significant interaction was found between the level of BNP and the presence or absence of diabetes. The OR for non-diabetic subjects was 1.26 (95% CI 0.61–2.60), but for diabetics the OR was 18.82 (95% CI 16.2–20.5). Tables 10 and 11. For cumulative rehospitalization within 12 months, an OR of 1.21 (95% CI 0.57–2.56) was obtained for non-diabetics, while for diabetics the OR was 18.7 (95% CI 16.5–20.8). The interaction *BNP x diabetes mellitus* could not be evaluated for other secondary outcomes due to lack of sufficient observations at each level of interaction.

Regarding BNP and atrial fibrillation, the adjusted OR was 3.88 (95% CI 1.45–10.38). Equally significant was the risk related to the occurrence of postoperative low cardiac output, with an OR of 3.46 (95% CI 1.53–7.80, p = 0.003). The other outcomes showed no significant association.

## Discussion

Our results show that high BNP is an important predictor of early and late postoperative complications. The OR adjusted for postoperative atrial fibrillation was 3.8 (95% CI 1.45–10.38). Several risk factors are associated with the occurrence of atrial fibrillation after cardiac surgery; they include mainly old age, low ejection fraction, and heart valve surgery. The onset of this arrhythmia is most likely a consequence of hemodynamic, electrical, and histological atrial tissue abnormalities related to intraoperative changes [20,21] and is associated with a higher incidence of early complications, such as congestive heart failure, stroke, renal dysfunction, infections, and neurocognitive impairment [22]. Postoperative atrial fibrillation increases hospital stays and health care costs [22,23], so strategies to predict its occurrence may have important clinical and economic relevance by intensifying preoperative medical treatment, enabling the use of perioperative intensive prophylaxis schemes, or eventually leading to the use of intraoperative ablation techniques. These advancements could decrease the incidence of adverse events in this high-risk group and reduce hospital stay, costs, and morbidity associated with atrial fibrillation [24].

The incidence of atrial fibrillation in our group was 11.2%, which is not as high as that reported in other series [22-24]; however, several subgroups at high risk for this complication had to be excluded. During their stay at general ward hospitalization it is also possible that some patients had transient episodes of atrial fibrillation that were not documented. This lack of detection, however, would suggest that these events did not have clinical significance.

The risk attributable to elevated BNP (cutoff for the top quintile: 258 pg/mL) in our group is very similar to that reported in the Cleveland Clinic series [25] that included 187 patients undergoing cardiac surgery, whose records were retrospectively evaluated, revealing an OR of 3.7 when comparing the highest with the lowest quartile. Our data also show that preoperative BNP measurement is valuable in predicting a significant increase in the probability of postoperative low cardiac output: OR 3.46 (95% CI 1.53–7.80); in turn, this complication is closely related to the overall morbidity and mortality [26]. The ability to anticipate the need for prolonged postoperative myocardial inotropy has important clinical implications, as the measurement of BNP can be used to identify patients who require intensive preoperative medical therapy. Additionally, a common problem in practice is to define the appropriate time to carry out cardiac surgery in patients who require complex procedures; perhaps BNP measurement could be useful in these patients. In this series, other unadjusted postoperative outcomes were also significantly related to high BNP: hospital stay, ICU stay, and number of transfused RBC units; each of these has been associated with increased postoperative morbidity and mortality. Our results open the possibilities of designing research studies that incorporate BNP measurement as a routine part of the preoperative evaluation and comparing this strategy with the standard evaluation, in terms of reducing postoperative adverse events.

Regarding late events, for the pre-specified primary outcome, a significant interaction was found between BNP and diabetes, with a significant increase in risk when BNP was elevated in diabetic subjects. This increase in risk among diabetic subjects with elevated BNP is most likely multifactorial in origin. Elevated BNP in diabetic subjects, with or without microalbuminuria, could represent the presence of an early stage of diabetic nephropathy, in which the level of creatinine is not yet affected [27,28]. It is possible that even mild degrees of renal dysfunction significantly impact postoperative morbidity. In the Steno-2 study of 160 type 2 diabetic subjects with microalbuminuria, higher baseline N-terminal-proBNP was associated with longer duration of diabetes, older age, higher systolic blood pressure, and impaired kidney function [29]. In our series, although diabetics tended to have higher morbidity and mortality at 12 months (primary outcome variable: 31.5% in diabetics vs. 29.1% in non-diabetics), this difference was not statistically significant. However, subjects with diabetes had other conditions associated with risk: older age (63.9 years vs. 59.0 years, p = 0.0001) and lower ejection fraction (50.0% vs. 52.6%, p = 0.017). These data could indicate that the subgroup of diabetic patients with elevated BNP have a higher comorbidity, which might explain our results. Moreover, diabetic heart disease is associated with a range of morphological changes, including myocyte hypertrophy, perivascular fibrosis, and accumulation of extracellular matrix amorphous protein. Over time, these processes contribute to the development of left ventricular hypertrophy, coronary artery disease, and congestive heart failure, each of which is associated with elevated BNP. Even if the ejection fraction is normal, occult cardiomyopathy characterized by impaired relaxation and a stiff left ventricle may precede manifestations of heart disease in patients with diabetes [30].

Finally, the hidden causes of nephropathy and more advanced heart disease may not be the only reason elevated BNP is associated with a significant increase in postoperative risk; other factors may include hypertension, increased extracellular volume, and pulmonary hypertension. Because all of these can be considered markers of cardiac risk, it is clear that the non-specific elevation of BNP can provide a useful indication of overall cardiovascular risk in this population.

Our data do not confirm that inflammatory markers were significantly and independently associated with the assessed outcomes; for hsCRP, the composite outcome had an OR of 1.53 (95% CI 0.80–2.91). For the same outcome, LEUCO had an OR of 1.41 (95% CI 0.78–2.55).

In a retrospective review of 720 patients undergoing cardiac surgery, where a measure of hsCRP was available, Cappabianca et al. identified that CRP ≥ 0.5 mg/dL conferred a higher risk of in-hospital mortality and postoperative infections [31]. Van der Harst, after evaluating retrospectively a subgroup of 87 patients with coronary revascularization, found that in patients with hsCRP above the median (1.9 mg/L), the cumulative incidence of cardiovascular events with a follow-up at 7.3 years was 29%, compared with 9% in patients with levels below the median (p = 0.048), independent of other risk factors [32]. However, Gaudino et al., in a prospective study of 114 patients undergoing myocardial revascularization surgery, found that CRP > 5 mg/L did not predict in-hospital postoperative complications or influence the extent of inflammatory activation [33]. In a larger series, Biancari et al. reported that preoperative CRP ≥ 1.0 mg/dL carried a higher overall risk of global postoperative death (5.3% vs. 1.1%, p = 0.001), cardiac death (4.4% vs. 0.8%, p = 0.002), and low cardiac output syndrome (8.8% vs. 3.7%, p = 0.01) (47). Ahlsson [34] found no association between preoperative CRP and postoperative atrial fibrillation.

The discrepant findings in the above results could suggest that multiple underlying risk factors are involved in the associations between adverse postoperative outcomes and hsCRP level. Insufficient adjustment for confounding variables related to the load of comorbidities may lead to an overestimation of risk. In our series, we carefully controlled for confounders by excluding active infectious and inflammatory or neoplastic processes. Furthermore, in multivariate analysis, age, gender, ejection fraction, diabetes mellitus, recent ACS, obesity, previous cardiac surgery, using or not using cardiopulmonary bypass, and type of surgery were controlled. It is possible that the relationship between preoperative and postoperative outcomes of hsCRP can largely be explained by multiple underlying factors that elevate hsCRP and increase surgical risk. However, because the ORs that we found for most of the outcomes were close to 1.5 for hsCRP throughout the follow-up, it is also possible that the sample size was insufficient to determine a real association.

Lower preoperative Hb was associated with increased postoperative adverse events in a Hb dose-dependent manner. A lower preoperative Hb (below 13.2 g/dL) was associated with increased in-hospital mortality and at 12 months and more likely to require inotropic drugs and suffer acute renal failure, cerebrovascular events, high-grade ventricular arrhythmias, and require RBC transfusion in the postoperative period. The primary composite outcome was also more frequently observed in the presence of lower Hb. Some pre-specified outcomes occurred entirely or almost entirely in the lowest hemoglobin quintiles (in-hospital mortality, acute renal failure, cerebrovascular events, and ventricular tachycardia); given their rarity and the fact that outcomes were concentrated in only one or two quintiles, we not considered it appropriate to test for statistical significance.

The effect of low Hb on most of the outcomes was attenuated after adjustment for the described covariates. However, the independent relationship between the lowest Hb values and postoperative low cardiac output was not affected by adjusting for covariates, yielding an OR of 0.33 (95% CI 0.13–0.81). A significant association was found between low preoperative Hb and major postoperative outcomes: hospital stay, ICU stay, and number of units of RBCs transfused. As mentioned above, these variables are associated with postoperative morbidity and mortality.

Reduced Hb may contribute to worse outcomes through a higher peripheral and myocardial oxygen demand and development of left ventricular hypertrophy, mainly due to a secondary increased cardiac output [35]. An inverse relationship between Hb value and left ventricular hypertrophy has been reported in clinical studies of patients with chronic kidney disease [36]. In a subgroup of patients with chronic heart failure, an increase of 1 g/dL in Hb was associated with a decrease of 4.1 g/m^2^ in left ventricular mass in a 24-week follow-up [37]. These changes could explain the increased risk of arrhythmias and the occurrence of low cardiac output in our patients with low Hb.

Our results show that the need for RBC transfusion in the perioperative period is directly related to the value of preoperative Hb, even in the absence of a defined anemia; subjects with Hb < 13.2 g/dL required an average transfusion of 3.1 units of RBCs, while if hemoglobin was > 16.1 g/dL, the required RBC units dropped to 0.6. One study showed that both preoperative anemia and intraoperative transfusion of red cells were independent and additive factors for adverse outcomes. Patients with low preoperative Hb had a higher incidence of postoperative adverse events, but for the same level of Hb, the risk of postoperative complications increased significantly with RBC transfusion. In addition, they observed a direct relationship between the number of RBC units transfused intraoperatively and the incidence of adverse events [38]. This independent association between RBC transfusion and adverse outcomes has been repeatedly described [39-44].

In this series, the fact that polycythemic subjects were at higher risk of postoperative adverse events could not be confirmed. However, this is physiologically plausible, and the number of subjects with this feature in the sample was low (16 patients); thus, it cannot be concluded that there was no such association.

Our findings open the possibility of designing randomized studies to assess if the optimization of preoperative Hb concentration, through the use of erythropoietin and/or iron therapy, would reduce perioperative transfusion and the incidence of ventricular arrhythmia, acute renal failure, and postoperative low cardiac output.

### Limitations

Although the sample size was calculated to detect an increase in risk by a factor of 2 for the variable Hb, the study may have been underpowered to detect the prognostic significance of hsCRP and LEUCO, most likely because inflammatory mechanisms are a weaker predictor of outcomes than Hb or BNP. The ORs found were slightly above 1.5; multicenter studies on a larger scale would be needed to achieve the required precision.

The incidence of atrial fibrillation may have been underestimated because continuous electrocardiogram monitoring is not routinely performed in postoperative patients after they are discharged from the ICU. Therefore, some patients may have had transient episodes of During their stay at general ward hospitalization. This lack of detection, however, would suggest that these events did not have clinical significance.

This study required multiple statistical analyses to assess the independent effects of four biomarkers on several postoperative outcomes. However, this does not increase the possibility of a type I error because the analysis of the data sets for each independent variable was done separately and all hypotheses were pre-specified.

## Conclusions

Our study reveals important risk factors that could help stratify patients undergoing heart surgery. Elevated BNP (top quintile: > 258 pg/mL) was independently associated with several adverse postoperative outcomes, especially atrial fibrillation, postoperative low cardiac output, and rehospitalization in the first year; the risk increased significantly in patients with diabetes mellitus. Low Hb (a dose-dependent effect for any value < 16.1 g/dL) was independently associated with the occurrence of postoperative low cardiac output.

Further studies are required to assess whether Hb or BNP level provides prognostic information in addition to the existing methods in terms of improving the prediction of postoperative adverse outcomes. The inclusion of these variables in risk stratification schemes could reduce morbidity and mortality, including hospital stay and costs of care.

### Definitions

Prespecified Definitions were used to assign precedents or outcomes, this way:

* **Ejection Fraction of the left ventricle** (EF): The last reported value of EF (before the surgery) in the echocardiogram or the left ventriculogram of each patient. When discrepancy was found in the result of these examinations, the EF was taken from the last of any of the two, before the surgery.

• **Previous cardiac surgery**: It is defined this way, if for the qualifying surgery (or current) there is the precedent in any stage of open heart surgery for any reason.

• **Reintervention due to bleeding**: New cardiac surgery, in the intrahospitalary period, after the accomplished qualifying surgery carried out for pericardial tamponade or excessive bleeding.

**Diabetes mellitus:** Any of the following situations: 1) A casual preoperative glicemia the same or greater to 200 mg/dl (11.1 mmol/l), glicemia in preoperative fasting same or grater to 126 mg/dl (7 mmol/l), 2) Regular ingestion of oral hypoglycemiants or regular application of insulin. 3) Previous diagnosis of diabetes mellitus carried out by a physician.

• **Obesity**: Body Mass Index> 30 Kg/m2

• **Surgical wound Infection**: Presence of inflammatory signs in the surgical wound associated with secretion in which bacterial growth is recorded.

• **Chronic pulmonary disease**: Diagnosis of Chronic Obstructive Pulmonary Disease (COPD) carried out by a physician and confirmed with espirometry which demonstrates a relation VEF1/CVF <70 % of what was predicted or findings on the thorax x-ray compatible with COPD symptoms of chronic cough and/or dyspnea.

• **Perioperatory acute myocardial Infarction (AMI)**: It refers to the AMI inside a 30 day postoperative (POP) period. Since the most frequent incidence is during the first hours of POP, in this period it is defined as the Segment Elevation located from ST in the EKG POP immediately followed by enzymatic elevation (Mb> 5 times upper limit and/or troponin I> 10 ng/dL at 12 hours of POP). Between the 3rd and 30th day POP, the diagnosis is identical to the one described in the following numeral (postoperative acute myocardial Infarction). Intubated Patients or with mental or neurological alteration that makes the evaluation of symptoms difficult, will be evaluated by a group of experts different from the investigators, to define if there is or not an AMI criteria.

• **Postoperative acute myocardial Infarction**: It refers to the AMI after the 30th postoperative day. It is defined as: Curve of ascent and descent of (Troponin I or T) biochemical markers of myocardial necrosis, with at least one of the following: Ischemic symptoms, pathological Q-wave development in the EKG, indicative EKG changes of ischemia (elevation or depression of ST segment).

**• Use of inotropes for more than 24 hours in postoperative (Postoperative low cardiac output)**: It refers to any inotrope administered intravenously in continuous infusion for more than 24 hours. For the specific case of Dopamine this criteria was considered to be positive if the dose was greater than 3 mc/Kg/min.

**• Acute Renal insufficiency**: Elevation of creatinine to values greater than 2 mgs/dL or increments of more than 1.5 mgs in regards to the preoperative value.

• **Cerebrovascular Event**: New neurological deficit, transitory or permanent, global or focal, with a duration of more than 24 hours and evidence of ischemic area in a cerebral computerized axial tomography (cerebral TAC) or magnetic resonance imaging (MRI). Psychomotor Deficits or patients diagnosed as "delirium", “ confussion” “ or “ demencia “ are not considered to have neurological deficit unless a neurological focal deficit is present.

• **Postoperative Atrial Fibrillation**: It was defined as an absence of P wave before QRS complexes together with irregular ventricular rhythm. A 12- Lead electrocardiogram was practiced to confirm the diagnosis.

• **Postoperative Ventricular Tachycardia**: 3 or more electrocardiographic beats of wide complex in the intrahospitalary follow up by means of continuous telemetry in the ICU.

• **Type of surgery**: 3 groups were considered: Group 1 formed by the myocardial revascularization surgery (CABG); Group 2, valvular substitution or repair surgery; Group 3 or combined, includes CABG related with valvular surgery with or without additional procedures, including ventricular restoration, surgery on 2 or 3 valves, ascending aorta replacement, etc.

## Abbreviations

Hb: Hemoglobin; LEUCO: Leukocyte count; hsCRP: High sensitivity C-reactive protein; BNP: B-type natriuretic peptide (BNP); ICU: Intensive care unit; PLCO: Postoperative low cardiac output; AF: Atrial fibrillation; ACS: Acute coronary syndrome; RBC: Red blood cells.

## Competing interests

The authors declare that they have no competing interests.

This study was funded mainly by a grant from Colciencias. Colciencias is a institution of government of Colombia which finances research projects. Abbott Laboratories of Colombia donated the reagents for BNP measurements for all patients. Except for the group of researchers, nobody had access to the cohort database. The study design, data analysis and conclusions have been carried out based entirely on the researchers ´ criteria.

## Authors’ contributions

EH directed all aspects of the study, including drafting the research proposal and submitting it to COLCIENCIAS, presenting and defending the protocol to the Institution´s Ethics and Research Committees, supervision of all the data collection of the study, review of the study´s CRFs and data base, and drafting of the final report. RD advised the methodological aspects of the study, strengthening the epidemiological and statistical tools; contributed to the drafting of the study´s written reports. DI contributed to the review of the possible outcomes and their definition as final events. JPU contributed commentaries to the manuscript and disseminated the objectives and scope of the research in the Institution. All authors read and approved the final manuscript.
